# Refillable silicone pump with precise switching for timed therapeutic delivery

**DOI:** 10.3389/fbioe.2025.1649771

**Published:** 2025-09-11

**Authors:** Naaz Thotathil, John J. Amante, Micah Wingell, Grace W. Hutter, Ultan Fallon, Yiling Fan, Keegan Mendez, Ellen T. Roche, Cathal J. Kearney

**Affiliations:** ^1^ Kearney Lab, Department of Biomedical Engineering, University of Massachusetts Amherst, Amherst, MA, United States; ^2^ Roche Lab, Department of Mechanical Engineering, Massachusetts Institute of Technology, Cambridge, MA, United States; ^3^ Roche Lab, Institute for Medical Engineering and Science, Massachusetts Institute of Technology Health Sciences and Technology Program, Harvard-Massachusetts Institute of Technology, Cambridge, MA, United States; ^4^ Wyss Institute for Biologically Inspired Engineering, Harvard University, Boston, MA, United States; ^5^ College of Medicine, Nursing and Health Sciences, University of Galway, Galway, Ireland

**Keywords:** soft robotics, on-demand drug delivery, refillable drug delivery device, finite element modeling, wound healing

## Abstract

**Introduction:**

Given the precise temporal coordination of natural biological processes, administering therapeutic agents at specific times can be used to enhance efficacy in a range of applications. To achieve such controlled drug delivery, various stimulus-responsive techniques (e.g., ultrasound, temperature changes, and electromagnetic radiation) have been developed. However, many of these current methods exhibit limitations, such as premature leakage prior to stimulus activation or delayed and prolonged responsiveness to stimuli. Our research introduces a soft robotic pressure-actuated drug delivery pump aimed at improving therapeutic efficacy through precisely-timed drug administration.

**Methods:**

This device utilizes silicone – a low-modulus material – for both the therapeutic reservoir and the actuation chamber to create a biocompatible and conformable interface, facilitating controlled drug release and offering the potential to be adapted as an implantable drug delivery system. Two ports in the actuation chamber allow the therapeutic reservoir to be refilled. We actuated the pressure reservoir of the device in the range of 28.5 – 59.8 mmHg and tested: the pressure-dependent release from the device; repeated release; baseline release, and the ability to deliver a wide-range of therapeutics.

**Results:**

Importantly, the system demonstrated a reliable On/Off mechanism – confirmed by actuating to ∼80% of opening pressure over 5 days – which addresses a key limitation in many existing technologies. In vitro, the device was used to deliver a range of therapeutics and had non-significant differences versus manual delivery of therapeutics in relevant assays: antibiotics (doxycycline; reduced E. coli viability by 49.6% vs. 49.8%); adeno-associated virus (AAV; transduced 73.5% vs. 76.2% of cells); dexamethasone (2D fibroblast scratch wound closure 50.9% vs. 51.0%); and successful delivery of viable cells (viability of 83% vs. 100%). We additionally developed a finite element model to model the pressure/volume release trend, and demonstrated the effect of membrane stiffness on release.

**Discussion:**

Our results demonstrate that the device can consistently administer therapeutics and molecules of various sizes and functions while maintaining their bioactivity, showcasing its potential for repeated, precisely-timed therapeutic delivery.

## 1 Introduction

Biological processes including wound healing, nerve repair, and fracture repair, all follow their own timed healing sequence (Peña and Martin, 2024; Dahlin, 2008; ElHawary et al., 2021). These processes are guided by growth factor signaling and cellular responses, and can be enhanced by delivering exogenous therapeutics (Bi et al., 2008; Ekenseair et al., 2013). When carefully timed, these therapeutics can be used to correct the aberrant natural signaling that causes delayed healing. For example, in the wound healing cascade, when timed steps are delayed due to external factors–such as stress, infection, disease–it can result in chronic wounds (Falanga et al., 2022). For these applications, and many others, timed drug delivery systems are being developed to overcome the limitations of conventional delivery methods (Adepu and Ramakrishna, 2021). These delivery systems have been shown to optimize therapeutic effects, enhance bioavailability, and help reduce the need for larger doses, minimizing side effects associated with off-target drug interactions (Zhao et al., 2020). In particular, stimulus-responsive drug delivery methods have been developed to further enhance control by incorporating triggering mechanisms for timed-control of delivery. These approaches leverage external stimuli, such as ultrasound, temperature, and pH to trigger the release of drugs at set times, dosages and locations (Bordbar-Khiabani and Gasik, 2022; Kohli et al., 2010). However, many of these systems rely on affinity or steric hindrance between the drug and carrier system and, thus, complete drug containment without any low level diffusion prior to release is a challenge (Song et al., 2020; Kubota et al., 2021; Kearney and Mooney, 2013). A second challenge is the ability to reload or restimulate these systems for multiple or longer-term therapy administrations, as temperature, pH, and ultrasound can cause material degradation and instability over time, impacting the long-term durability of these systems (Hibbins et al., 2017; George and Abraham, 2007).

A more recent approach in biomedical engineering involves soft robotic systems. The field of soft robotics integrates low-modulus materials into a system to create a biocompatible interaction with the human body (Lee et al., 2020; Barata et al., 2013). Soft robotics can be precisely controlled and manipulated, allowing for controlled drug release rates, dosage levels, and repeated administration at user-defined times (Whyte et al., 2022; Wallace et al., 2025; Park et al., 2024). Additionally, unlike traditional rigid robotic systems, the ability of soft robots to be positioned in a dynamic environment allows this technology to have the capability to achieve targeted delivery in a wider range of anatomical locations such as the stomach, heart, and peritoneum (Eshaghi et al., 2021; Cianchetti et al., 2018). To date, soft robotics have been explored as ingestible, semi-implantable, or implantable drug delivery systems (Srinivasan et al., 2022; Zhou and Alici, 2022; Heunis et al., 2023; Yim and Sitti, 2012; Beatty et al., 2023; Iacovacci et al., 2021). Each robotic system aims to induce controlled and targeted delivery by being retained at the tissue site, providing tissue specific delivery (Zhou and Alici, 2022; Zhang et al., 2015).

There exists a wide range of therapeutics that would be more effective if they were delivered at the correct time (Mirvakili and Langer, 2021; Brudno and Mooney, 2015). To demonstrate the flexibility of our system, here, we chose to explore a diverse selection of molecules that can potentially benefit wound healing by being delivered at the appropriate time and maintain bioactivity. These include doxycycline, adeno-associated virus (AAV), and dexamethasone. Doxycycline, a small molecule tetracycline antibiotic, is used to treat infections, which are a common challenge in wound healing, particularly in chronic wounds. Doxycycline has also been reported to have additional wound healing benefits, such as anti-scarring (Moore et al., 2020). Additionally, gene therapy is an increasingly explored approach to treat difficult to heal wounds (Shaabani et al., 2022; Karimzadeh et al., 2024). While several viruses are being researched, AAV has emerged as one of the preferred vectors for viral delivery to skin as it is non-integrating and has proven efficacious in pre-clinical models (Agrawal et al., 2004; Ain et al., 2021). Finally, our lab has become increasingly interested in the role of circadian rhythms in wound healing. Circadian rhythms are the natural endogenous 24 h cycles that our bodies and cells experience daily; these rhythms are known to play central roles in many biological processes, including skin wound healing (Jacob et al., 2020; Hoyle et al., 2017). To study these rhythms *in vitro*, cells need to be carefully synchronized or entrained to a rhythmic signal. Dexamethasone–a glucocorticoid–can be used to synchronize circadian rhythms of cells in in vitro cultures (Pagani et al., 2010; Izumo et al., 2006; Yang et al., 2022).; when dexamethasone is applied to 2D wound scratch assays, the improved wound closure is attributed to circadian rhythm synchronization (Ngo et al., 2019; Beta et al., 2022; Tu et al., 2020; Nakazato et al., 2023).

Here, we focused on a non-implantable device for delivery to wounds due to our longer-term interest in chronic wound healing models. Previously, we reported on a similar soft robotic drug delivery device for local epicardial drug delivery, achieving a reliable On/Off drug release system in response to epicardial ECG using pressure actuation demonstrating the ability of our device to be made implantable (Mendez KL. et al., 2024; Mendez K. et al., 2024). The previous device was manufactured using lost wax casting to generate an actuation element and a drug chamber (Mendez KL. et al., 2024). Following unidirectional actuation and expansion, drug is precisely ejected through a single pore. We demonstrated that by adjusting manufacturing parameters (e.g., elastomer stiffness, pore size, membrane pre-stretch) and/or actuation parameters (e.g., actuation volume, number of cycles), we could tune the amount of drug released (Mendez KL. et al., 2024). Fatigue testing of these devices up to 10,000 cycles at 62.1 kPa did not show any drop off in mechanical reaction, and when subsequently tested for burst pressure, the devices reached a pressure of ∼275 kPa (Mendez KL. et al., 2024).

In this iteration, we adapted the system to connect the drug chamber to a refillable port. The overall objective of our study was to investigate the refillable silicone pump’s ability to release consistent dosages only in response to pressure-input. We additionally tested whether the device could release a range of therapeutics without adversely affecting their efficacy. We show that the pump can deliver a wide range of drugs from small molecules to cells, while still maintaining their bioactivity.

## 2 Materials and methods

### 2.1 Characterization of silicone refillable device

#### 2.1.1 Device manufacturing

Devices were manufactured as previously described (Mendez KL. et al., 2024). Briefly, lost wax casting was used to manufacture the actuation element, composed of high stiffness (Smooth-Sil 950; modulus = 1,800 kPa, tensile strength = 5 MPa) in the pressure reservoir, and low stiffness silicone (Ecoflex 00-20; modulus = 55 kPa, tensile strength 1.1 MPa) in the drug reservoir to enable unidirectional actuation expansion. The self-sealing drug reservoir membrane had a single pore created using an 18G hypodermic needle, and two ports to allow for evacuation of the chamber and drug filling.

#### 2.1.2 Device setup, connection and actuation

**FIGURE 1:**
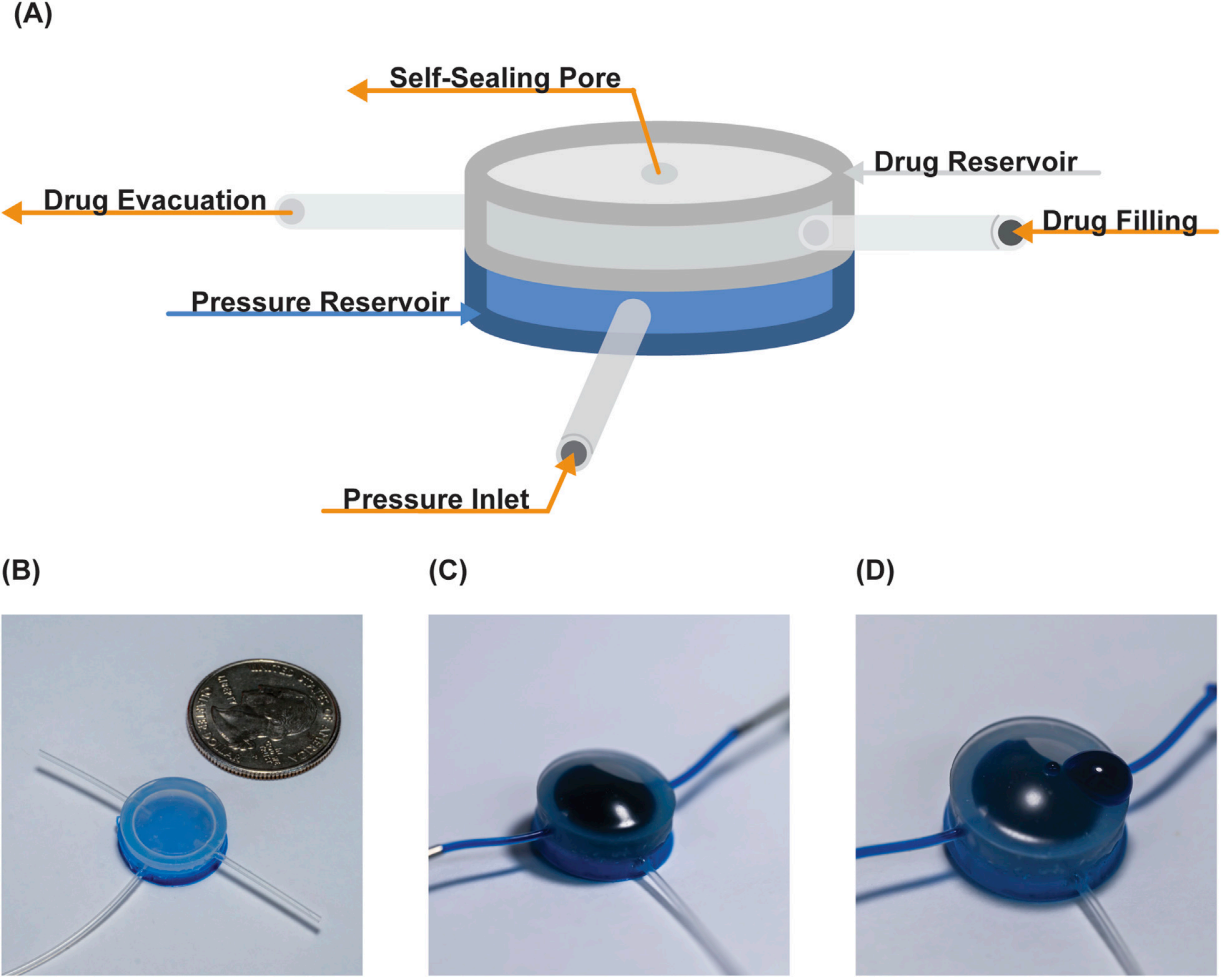
Overview of pump. **(A)** Schematic identifying the two reservoirs and their connections. Photographs of the device **(B)** post-manufacturing, **(C)** drug loaded, and **(D)** following drug release.

For each of our release studies, we used the same device setup. The device consists of two reservoirs: an actuation reservoir and a therapy reservoir. The actuation reservoir (Figure 1) is linked to a syringe for pressure modulation (‘pressure inlet’ tube in Figure 1), with the syringe attached to a syringe pump (New Era Pump Systems Inc. #1000) and pressure gauge (Pasco, model PS-3202). The therapy reservoir has two ports: one port (therapy evacuation line) is used to remove air from the chamber to create volume for filling, and the second port (therapy filling line) is used to fill the therapy reservoir chamber. Following loading, the reservoir is sealed off using luer lock valves. This setup facilitates the drug loading, control, and subsequent release of the drug from the device by adding air to the pressure reservoir through the pressure inlet at select volumes, which were measured as pressures (28.5–59.8 mmHg; 200–600 µL actuation volumes; 400 µL = 58 mmHg actuation pressure).

To characterize the device, we utilized a known concentration of food dye solution to establish a standard curve to determine volume release in response to pressure. Following a 150 µL volume loading of the therapy reservoir, a syringe pump was used to increase the pressure of the actuation reservoir. These pressures were read by an in-line pressure gauge which identified the pressure range (28.5–59.8 mmHg) for release, and this range was used to trigger release into 1 mL of water. To confirm the absence of leakage between release, we initiated release from the pump at 28.5 mmHg, returned the pressure to 0 mmHg, and then triggered the device once more at 28.5 mmHg. Next, we actuated the device at 28.5 mmHg, 50 mmHg, and 59.8 mmHg (returning to 0 mmHg in between each actuation) to yield three approximately equal partial releases from a single 150 µL fill of the therapy reservoir. To assess the consistency of release across multiple repeat actuations and refillings, we conducted a release assay of 12 consecutive releases at a pressure of 59.8 mmHg.

#### 2.1.3 Robustness testing

To analyze the robustness of the device after repeated cyclical actuation we performed a release/refill test (*n* = 12 cycles; 1,000 μL actuation with water), subjected the device to 100 complete actuations (1,000 μL) with water, and then repeated the release/refill testing (*n* = 12 cycles; 1,000 μL actuation with water). Before the test, air was evacuated from both chambers using a syringe. The drug chamber was filled with a fluid containing dye as a drug analog. A stopcock was used to fill the chamber with fluid until it reached pressure equilibrium. Water was used for the actuation chamber. The actuation pressure was applied, drug analog released, and then a stopcock was used to open the system to a reservoir of drug analog for refill. A scale (Sartorius LA120S) was used to measure the release volume. This was repeated 12 times with drug analog in the drug chamber (pre actuation), then 100 times without drug analog (cycling the actuation chamber), and another 12 times with drug analog (post actuation). Care was taken to ensure the membrane was in a neutral position at the start of each experiment.

#### 2.1.4 Baseline release testing

To assess baseline leakage from device we suspended devices containing 10 mg/mL acid red one solution in 20 mL of deionized (DI) water. The devices remained in the solution for 19 h before the first sampling. Then, we sampled 200 μL before and after daily actuation for 5 days, replacing the same volume of DI water after each sample. Devices were actuated in a separate vial to a target pressure of 25 mmHg using air, ∼80% of the device’s opening pressure, and the media sampled. Samples were read on a plate reader at an absorbance of 506 nm and a standard curve was used to calculate the released volume. Two devices were excluded from the experiment due to artifact dye (from luer lock and needle) in the test sample.

### 2.2 Finite element model for pressure-volume characterization, and device parameter tuning

We conducted a finite element analysis to simulate the device actuation at various pressures, and calculate the corresponding drug release volume, demonstrating refill from a larger reservoir. Abaqus (Dassault Systemes) was used for the analysis with C3D4, four node linear tetrahedron elements (total number of elements was 239,548. The global element size was 0.3 with a refined mesh around the pore (5 seeded elements). The geometry is shown in Figure 3A, and we used a hyperelastic constitutive model. In a series of simulations, we applied an actuation pressure of 40, 50, 60, 70, and 90 mmHg at the deflecting membrane at the base of the drug chamber, causing it to deflect. The simulation failed at 120 mmHg. At the experimentally defined critical opening pressure (see 3.1), we simulated drug release by decreasing the fluid viscous coefficient in a pressure dependent manner (from 1E8 to 400), so that fluid could escape from the pore at a predefined pressure. The interaction property viscous coefficient was set to 450 between the reservoir and the device. Upon fluid release the pressure in the chamber decreases, the pressure load is removed and the membrane deflects back to baseline, drawing fluid in from the reservoir to refill the chamber. To simulate different stiffnesses, the first coefficient of the hyperelastic model was halved, and doubled, and the simulation was repeated.

### 2.3 Bioactivity studies

#### 2.3.1 Small molecule antibiotic release

Competent *Escherichia coli* (*E. coli*) were combined with Luria Broth (LB broth) and allowed to grow in a shaker at 200 rpm and 37 °C for 24 h. Following incubation, e suspension was dispensed into individual wells of 24 well plates and biofilms were allowed to form over 24 h. To determine the ability of our device to release antibiotic, three experimental groups were established: a no treatment group (negative control); a positive control group, where doxycycline (30 μL at a concentration of 1 μg/mL) was manually administered; and a Pump Release group where doxycycline was released from the pump (approximately 30 μL at a concentration of 1 μg/mL doxycycline). Two hours post-antibiotic exposure, Alamar blue (Gibco) was added to the samples to enable the quantification of live cells via fluorescent intensity. Films were imaged in bright field.

#### 2.3.2 Bioactivity of adeno associated virus

HEK293T cells were cultured in Dulbecco’s Modified Eagle’s Medium (High Glucose, L-Glutamine, Sodium Pyruvate) supplemented with 10% Fetal Bovine Serum and 1% Penicillin- Streptomycin. eGFP-AAV (e-GFP = enhanced Green Flourescent Protein) — an adeno associated viral vector that transduces cells to express the green fluorescent protein (VectorBuilder) was used as a model viral vector. When HEK293T cells were cultured and grown to reach a density of 90,000 cells per well, a multiplicity of infection of 1 (MOI 1, i.e., 1 AAV infects each cell on average) was introduced per well using either direct addition or released from the pump. Forty-eight hours following viral administration, the expression of eGFP within the cells was quantified using flow cytometry (LSRFortessa three laser), enabling the assessment of cell transduction. Subsequent analysis of the flow cytometry data was conducted using FlowJo software and sample gating is presented in Supplementary Figure S1.

#### 2.3.3 Dexamethasone delivery in a wound scratch assay

BJ fibroblasts (ATCC, CRL-2522, primary cells) were cultured in Eagle’s Minimum Essential Medium (ATCC) supplemented with 10% Fetal Bovine Serum and 1% Penicillin- Streptomycin. On day 1, fibroblast cells were cultured and seeded at 90 k cells per well in a 6-well plate. On day 5, ∼50 µL dexamethasone stock was released from the pump or delivered manually to give a final concentration of 100 nM dexamethasone in treatment groups. The dexamethasone pulse was left in the wells for 60 min to induce circadian synchronization (Pagani et al., 2010; Izumo et al., 2006; Yang et al., 2022; Ngo et al., 2019; Beta et al., 2022; Tu et al., 2020; Nakazato et al., 2023). Following an 18-h incubation, a vertical scratch was performed with a 200 µL pipette tip. Images were taken immediately after scratch and 12 h later to assess fibroblast regrowth, and the wound area quantified using ImageJ software.

### 2.4 Biocompatibility test

#### 2.4.1 Silicone pump interfaced with BJ fibroblasts

To assess the biocompatibility of the device, we conducted a viability test on fibroblast cells. Initially, 90 k fibroblast cells were seeded on day 1 in a 6-well plate. On day 3, sterilized pumps were introduced to float over the media. After 24 h, the viability of the cells was quantified using trypan blue (Gibco) with a cell counter (Accuris Instruments Quadcount).

#### 2.4.2 Fibroblast release test from silicone pump

A total of 90 k fibroblast cells were loaded into the pump and released into media-filled wells in a 6-well plate, estimating release of approximately 30 k cells per well. Images were captured every 12 h over a span of 48 h to monitor growth progression. At 48 h, the quantity and viability of cells was measured using trypan blue (Gibco) and a cell counter (Accuris Instruments Quadcount).

### 2.5 Statistical analysis

The data were graphed as mean ± standard deviation using GraphPad (Prism 10.0) unless otherwise described. For studies with three groups (e.g., no treatment, Positive Control, Treatment with Pump), one-way ANOVA with Holm-Sidak’s multiple comparisons test was used. If cells were treated in two independent groups (e.g., cell viability), an unpaired t-test was used. In graphs, significance is labelled as: * = p < 0.05, ** = p < 0.01, *** = p < 0.001, and **** = p < 0.0001.

## 3 Results

### 3.1 Characterization of silicone refillable device

**FIGURE 2:**
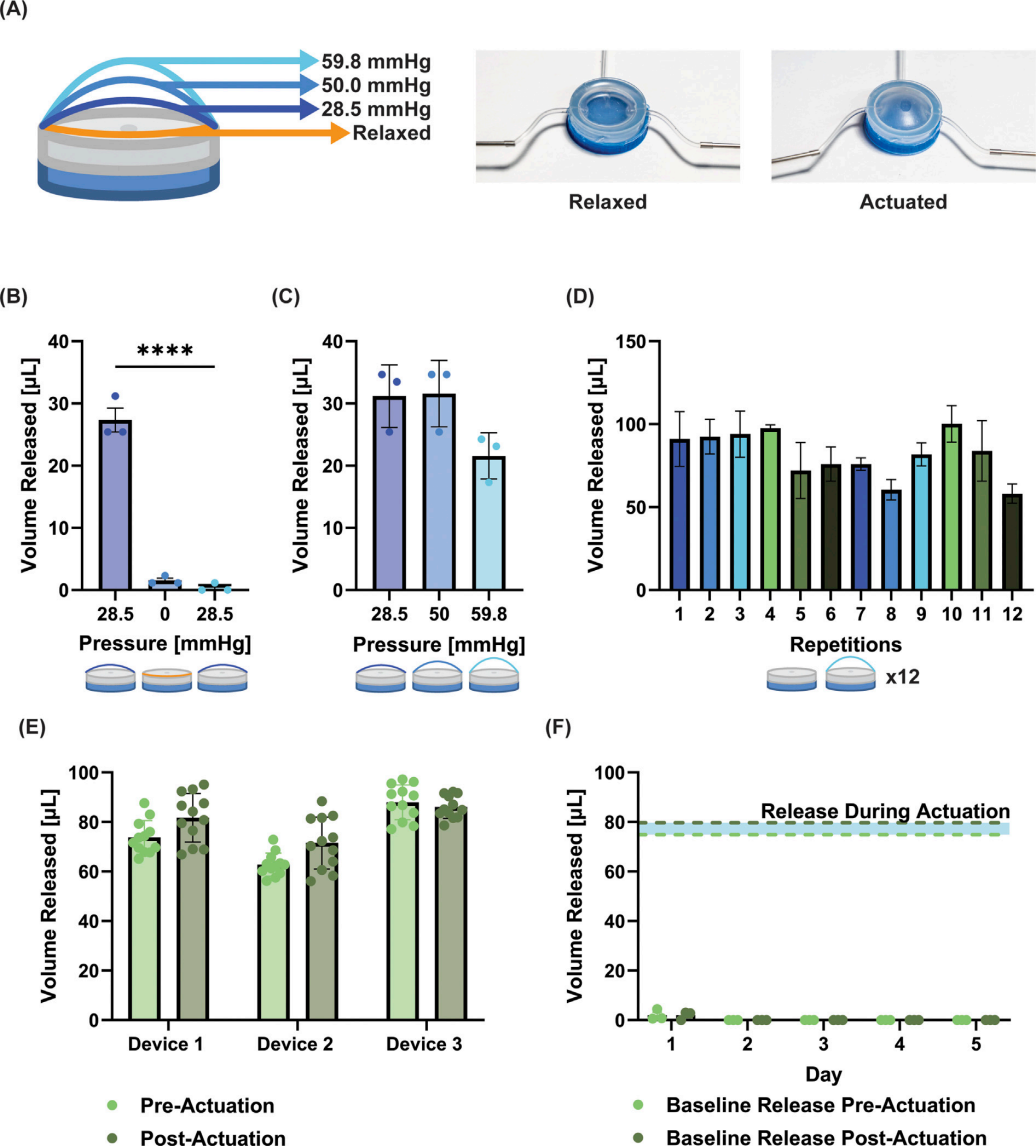
Characterization and optimization of silicone pump. **(A)** Images of device in the relaxed and actuated state. Schematic shows the increasing actuation at defined pressures. **(B)** Volume released when the device is triggered at a set pressure (28.5 mmHg), returned to zero pressure, and re-actuated at 28.5 mmHg without refilling. Between actuation values and following re-actuation, no leakage from the device is observed. **(C)** Volume released at three increasing pressures (the pressure is reduced to zero between actuations) from one single 150 µL fill of the device. (no significant difference observed). **(D)** Repetition of release at 58.9 mmHg with refilling between demonstrates consistent release up to 12 repeats. **(E)** Release volumes from device pre- and post-cyclical actuation (100 cycles). The release/refill testing was run for 12 cycles before and after cyclical actuation (no significant differences observed between pre- and post-actuation groups for any device). **(F)** Baseline release volume pre- and post-actuation to 25 mmHg (∼80% actuation pressure) for 5 days; for reference the blue shaded area demonstrates the volume released during actuation. While a small amount of release was observed on day 1, all subsequent days had zero values. No significant differences were observed between pre- and post-actuation. Data is presented as mean ± standard deviation, **** = p < 0.0001.

The device was manufactured to simulate a unidirectional actuation expansion, which can be observed in photographs of the device (Figure 2A). With each increasing pressure trigger, the drug loaded chamber enlarges and when a critical opening threshold is reached drug release occurs. With 150 µL volume therapeutic loading, it was established that the release is initially triggered at 28.5 mmHg, with complete release occurring between 28.5 mmHg and 59.8 mmHg, leaving residue of ∼50 µL in the drug chamber. When the pump was pressure triggered at 28.5 mmHg with a singular 150 µL fill, there was an average volume release of 27.4 µL ± 3.4 µL (Figure 2B). There was no visual evidence of opening of the pump or release at 0 mmHg or when the device was re-actuated back at 28.5 mmHg; the readings at these values were within the fluctuations of the blank values for our plate reader (1.50 µL and 0.37 µL for 0 and 28.5 mmHg, respectively) (Figure 2B). This demonstrated that the pump would not release without a pressure increase beyond the original trigger point. To demonstrate the ability to release volumes within the range of pressures identified for full release (28.5 mmHg–59.8 mmHg), we tuned the pressures to get approximately equal releases at increasing pressures. At 28.5 mmHg, 50 mmHg, and 59.8 mmHg, the volumes released were 31.3 ± 5.7 µL, 31.7 ± 6.3 µL, and 21.4 ± 5.5 µL (when analyzed with One-way ANOVA these differences were not statistically significant) (Figure 2C). Finally, to assess the device’s repeated-release consistency, we measured the release volume at 59.8 mmHg stimulation for up to 12 consecutive releases, with refilling between each pump. This resulted in an average release volume of 82 µL ± 14 µL (when analyzed with One-way ANOVA the differences between repeated actuations were not statistically significant) (Figure 2D). We believe that the variations among test groups is due to fluctuating residual volume within the device over repeated cycles. Drug filling could be more consistently controlled by more accurately controlling filling pressure, as opposed to volume as done here. Thus, as part of the subsequent robustness testing, we used a pressure-controlled strategy to ensure residual volume was accounted for.

### 3.2 Robustness and baseline release testing

The results (Figure 2E) show that the device (*n* = 3) could release the same volume for 12 cycles, was robust to multiple cycles of actuation (*n* = 100), and could release the same volume after cyclical actuation for a further 12 cycles (averages pre–and post–actuation for each device = 73.7 and 81.7 µL; 62.8 and 71.5 µL; 88 and 86 μL; no significant differences between pre- and post-actuation readings). With this strategy to account for equilibrating pressure during refilling, the standard deviations appeared to be reduced for the devices (±6.7, 4.7, 7.0 µL for each device).

When devices were actuated to ∼80% of their opening pressure for five consecutive days, we observed 2 µL of release during the first 19 h of incubation, and 1.7 µL of release following the first actuation (Figure 2F). We note that these readings were just above the sensitivity of our plate reader. Subsequently, release readings were all zero for each reading pre- and post-activation up to the 5 days tested, suggesting a very robust On/Off mechanism for our device.

### 3.3 Finite element model for pressure-volume characterization, and device parameter tuning

**FIGURE 3:**
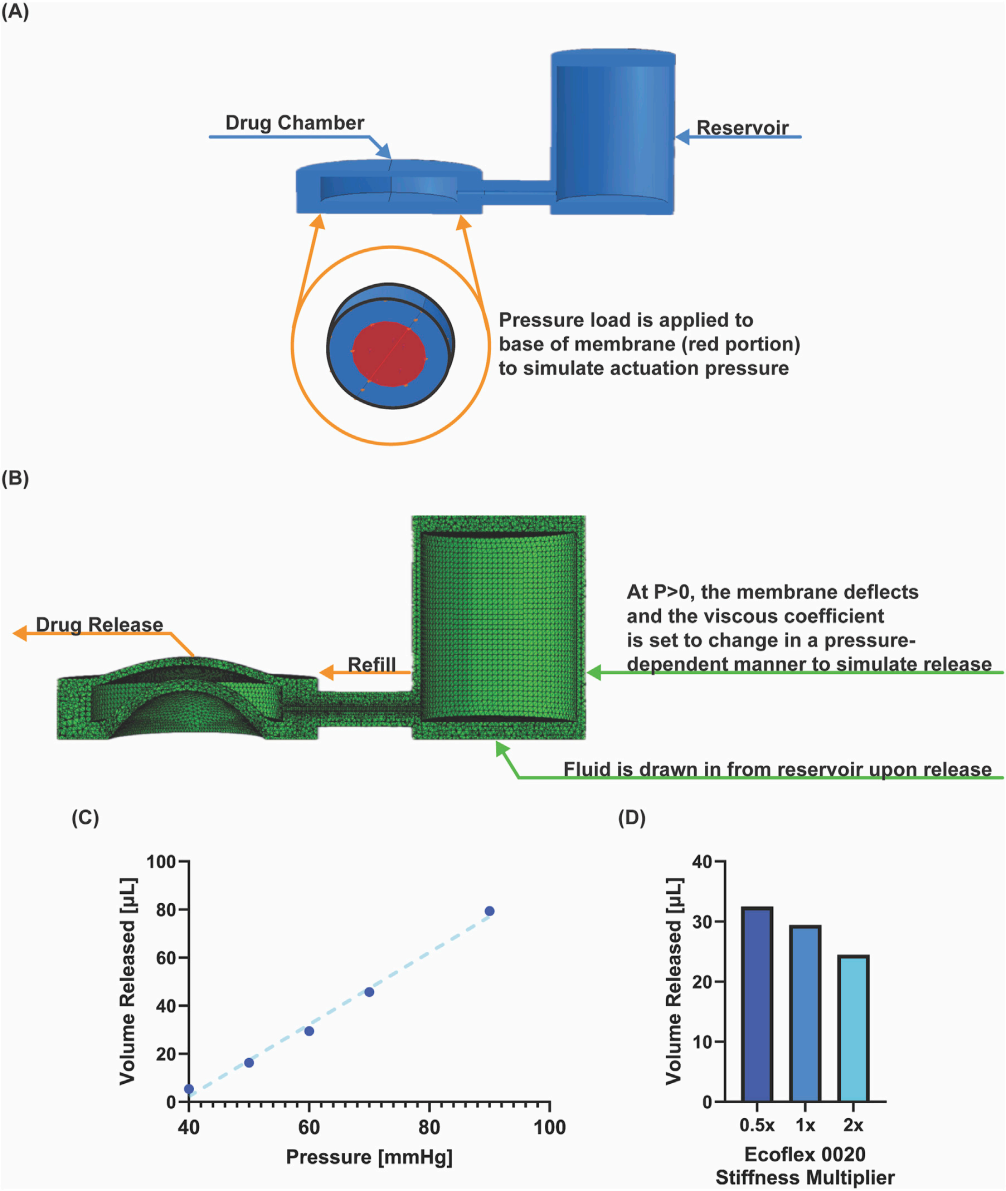
Finite element model of device. **(A)** Set up of geometry and pressure load application. **(B)** Deformed structural model during pressure application. **(C)** Pressure-volume relationship from six simulations at indicated pressure. **(D)** Simulated volume released for changing stiffness, implemented by halving and doubling the first coefficient in the hyperelastic model.

The pressure-volume characterization (Figure 3) shows the pressure/volume release trend. Higher actuation pressures resulted in a higher release volume, with a linear trend (Figure 3C). Increasing the stiffness of the membrane–while maintaining a constant actuation pressure–results in less drug released (Figure 3D). Dimensional and material parameters can be easily varied for optimizing design of the device for a given application. Having established the device’s ability to repeatedly dose a consistent/controlled volume from the device, we next wanted to test the device *in vitro* in a range of specific biological assays that could have applications in wound healing.

### 3.4 Bioactivity of antibiotic released from pump

**FIGURE 4:**
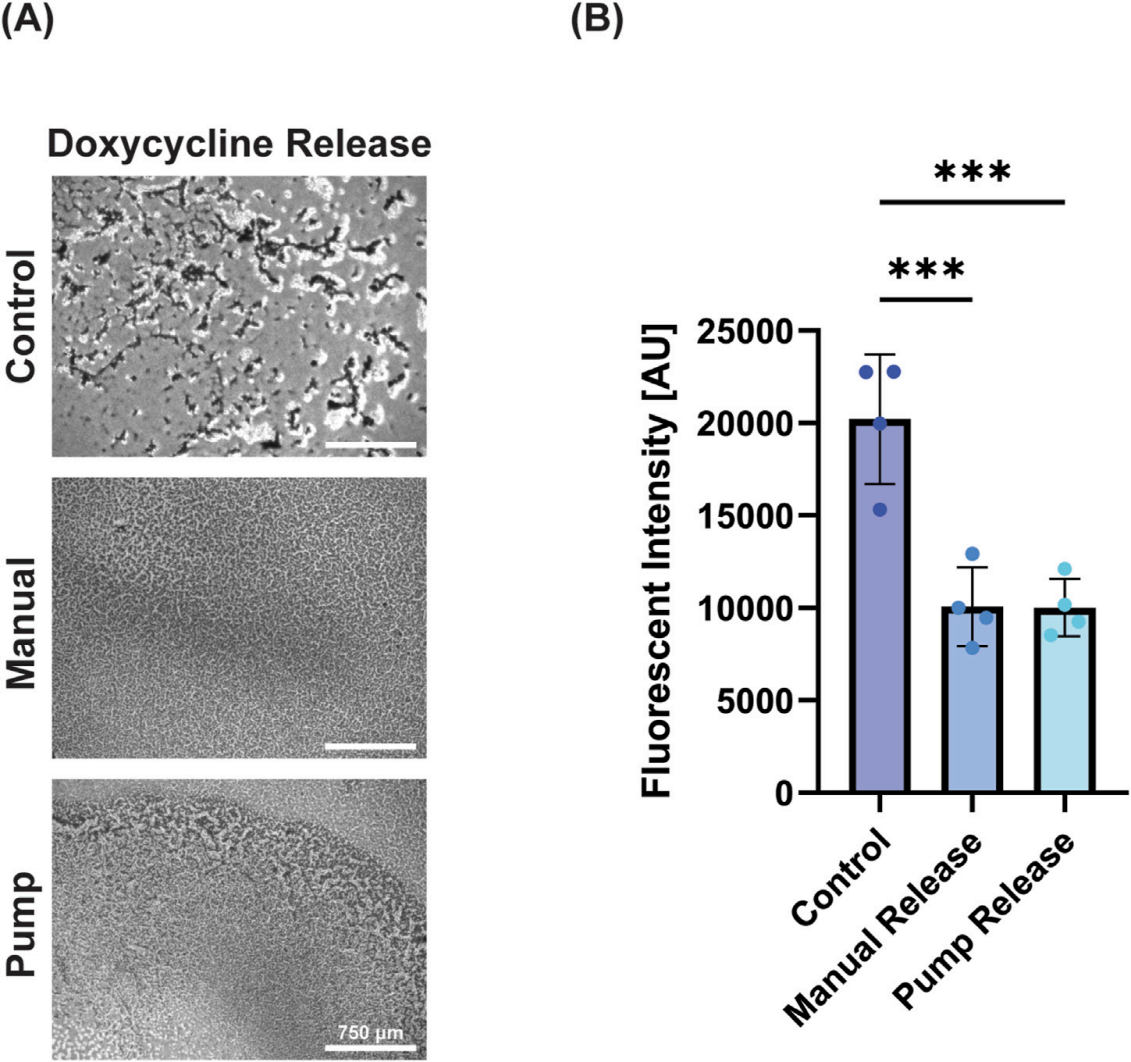
Antibiotics successfully retained bioactivity. **(A)** Microscope images of *E. coli* with no antibiotic release, manual release of antibiotic, and pump release of antibiotic (left to right) demonstrating reduction of bacterial load with doxycycline treatment (scale bar = 750 μm). **(B)** Percent viability of *E. coli* (as measured by Alamar blue) following treatment demonstrating the efficacy of pump-released antibiotics. Data is presented as mean ± standard deviation, *** = p < 0.001.

As infection is a common challenge in wound healing, we next wanted to determine the ability of the device to deliver a small molecule antibiotic, doxycycline. Doxycycline was released onto *E. coli* biofilms resulting in decreased viability for both treatment groups (Figure 4A). The manual doxycycline and pump doxycycline groups reduced viability near-identically (49.8% and 49.6%; no significant difference), demonstrating the pump’s ability to deliver a bioactive dose that matches its manually delivered counterpart (Figure 4B). The device reliably delivered the desired antibiotic dose resulting in expected bacterial death matched to controls.

### 3.5 Bioactivity of adeno associated virus released from pump

**FIGURE 5:**
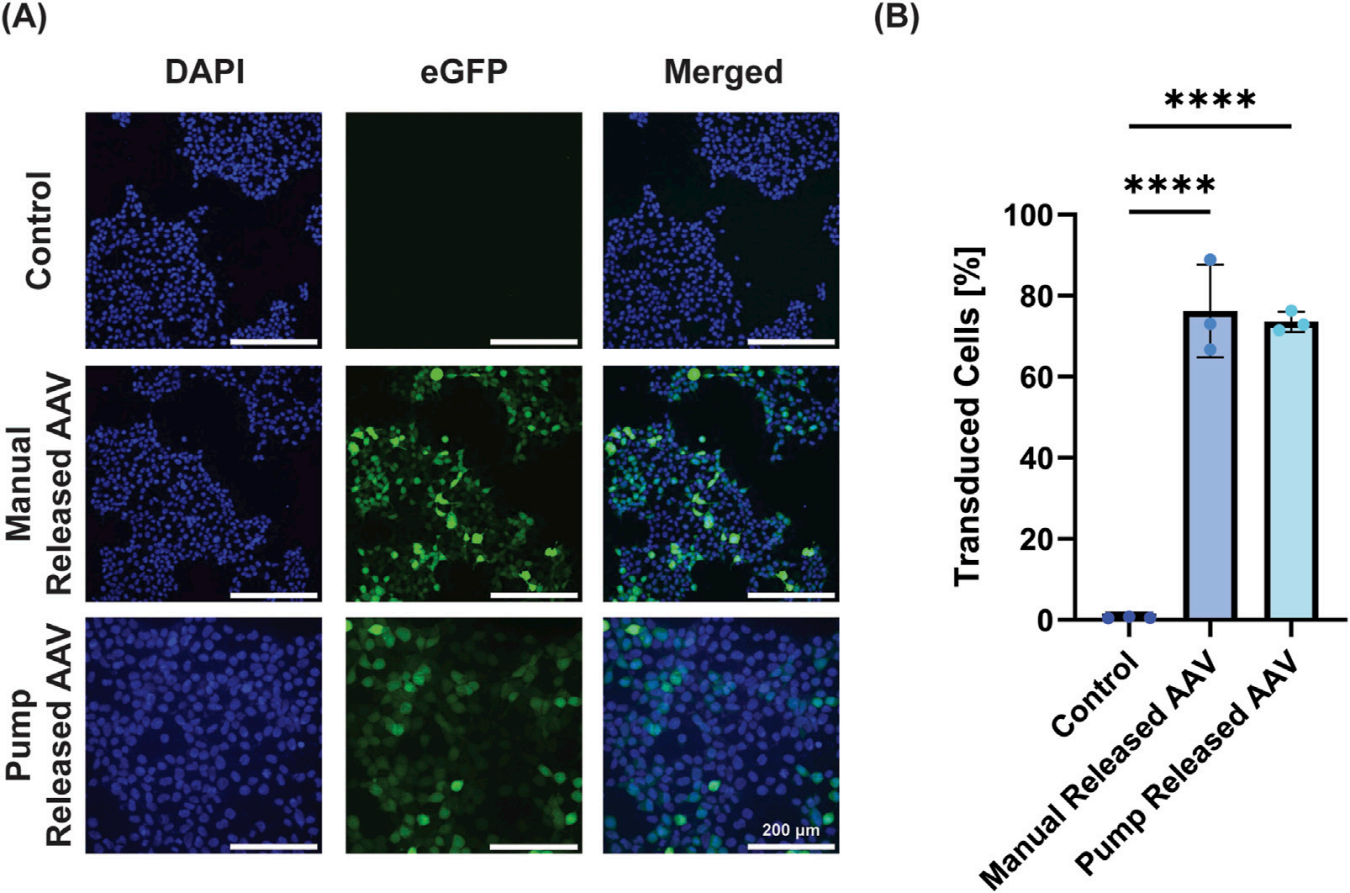
Pump-released virus successfully transduces cells. **(A)** Representative images demonstrate that groups treated with AAV-eGFP (50 µL AAV-eGFP at MOI = 1) directly added or released from the device transduce HEK293T cells to express eGFP (DAPI, cell nuclei; GFP, green channel; merged, overlay of both channels; scale bar = 200 µm). **(B)** Quantification of GFP^+^ cells using flow cytometry demonstrates equal efficacy for manual and pump AAV-eGFP treatment (data is presented as mean ± standard deviation, **** = p < 0.0001).

Next, the ability to release a biologically active virus was performed with AAV-GFP. AAV-GFP is often used as a model system to demonstrate transduction potential of a system prior to including therapeutic genes. Here we successfully transduced HEK293T cells with AAV-eGFP (MOI = 1) by both standard manual addition of virus and using pump release of the virus (Figure 5A). When quantified using flow cytometry, the manual control transduced 76.2% ± 11.4% of cells, while the pump transduced 73.5% ± 2.5% (Figure 5B). While both groups were significant versus negative control, there was no significant difference between the manual and pump administration, demonstrating the ability of the pump to successfully release AAV.

### 3.6 Bioactivity of circadian synchronizing molecule released from device

**FIGURE 6:**
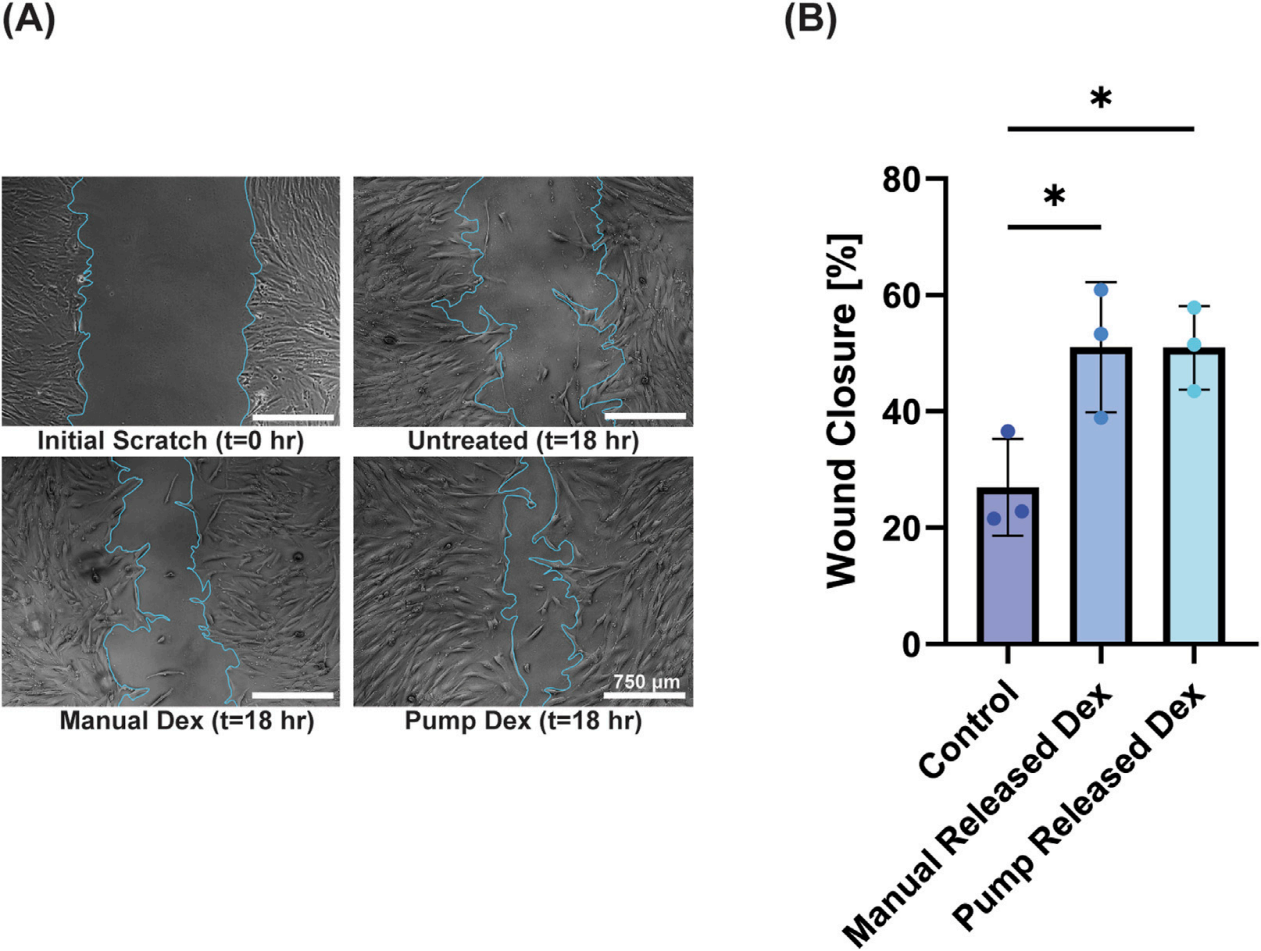
Pump delivery of dexamethasone to fibroblasts enhances wound healing in a 2-D scratch assay. **(A)** Images of 2-D scratch assay of fibroblasts immediately post-wounding (t = 0 h) and at 18 h with (manual dex, pump dex) and without (untreated) dexamethasone treatment (scale bar = 750 μm). **(B)** Quantification of wound closure at 18 h, demonstrating the advantage of dexamethasone addition (mean ± standard deviation; * = p < 0.05).

While each individual cell in a culture has a circadian rhythm, they are not in synch with other cells in the dish; hence, effects of circadian rhythms are averaged out across the culture in standard conditions. As circadian rhythms play key roles in many biological processes, inducing synchronized rhythms in culture is key to understanding biological processes in more representative models. One drug that can accomplish this is dexamethasone (Dex), a synthetic glucocorticoid that acts like cortisol and facilitates the synchronization of cellular circadian rhythms. Dex has shown to help align biological activities across several cell types, enhancing coordinated responses that are effective in wound healing (Beta et al., 2022; Tu et al., 2020). As the precise timing of Dex delivery is critical, using the pump we demonstrated that a single pulse of Dex was able to enhance wound healing in a 2D BJ Fibroblast scratch assay (Figure 6A). Whereas unsynchronized cells closed wounds by 27.0%, both manual delivery of dexamethasone and pump delivery of dexamethasone significantly enhanced healing versus non-Dex treated controls (51.0% ± 11.2% and 50.9% ± 7.2%, respectively) (Figure 6B). While potential direct effects of dexamethasone cannot be ruled out here, our finding is consistent with others that attributed enhanced wound closure following dexamethasone pulsing to circadian rhythm synchronization (Ngo et al., 2019; Beta et al., 2022; Tu et al., 2020; Nakazato et al., 2023).

### 3.7 Biocompatibility of silicone device

**FIGURE 7:**
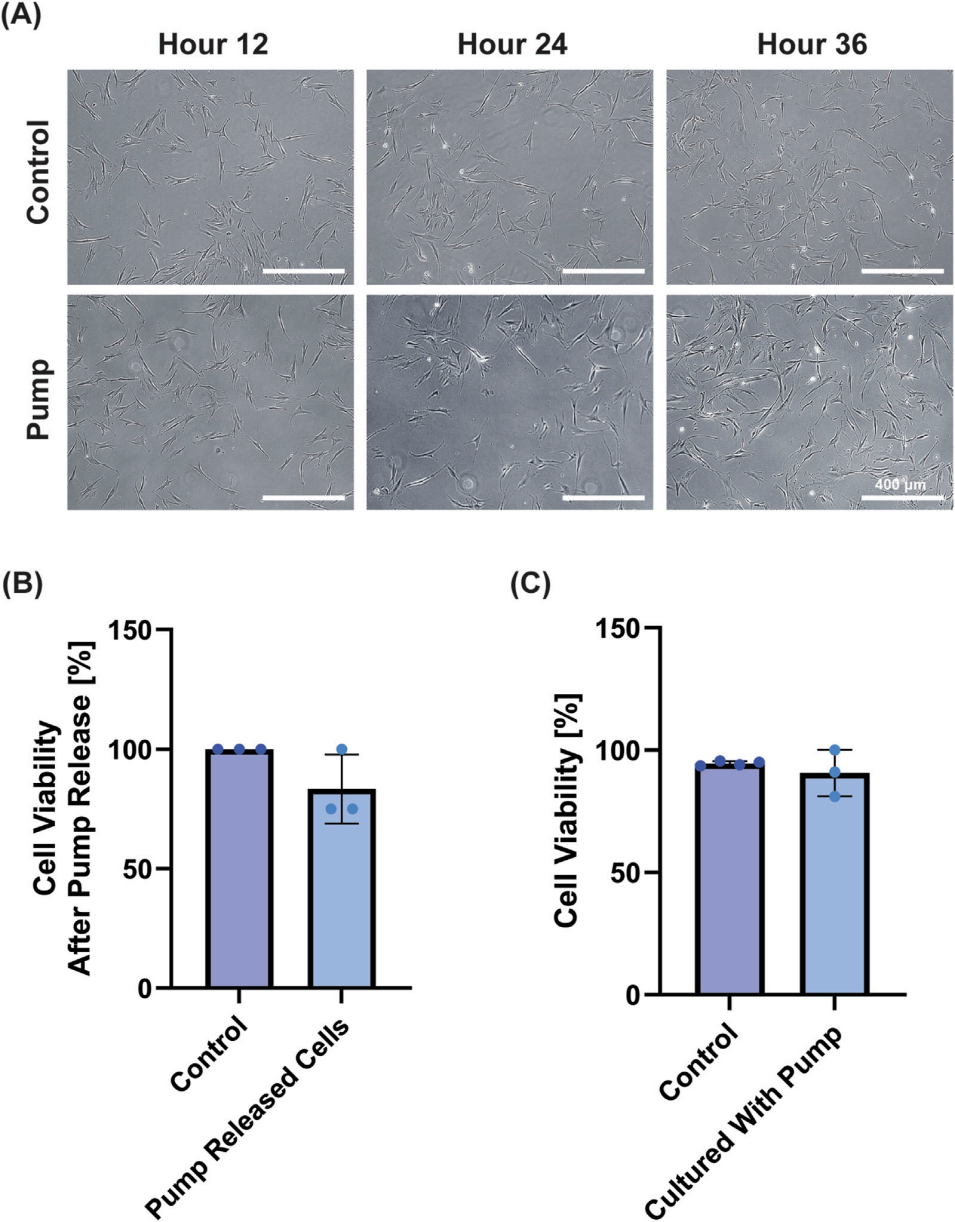
Biocompatibility Test with fibroblasts. **(A)** Representative images demonstrate cell growth after 12, 24 and 48 h for the control and treatment group after cells were loaded into and released from the pump (Scale bar = 400 μm). **(B)** Cell viability of the cells measured at 48 h demonstrated BJ Fibroblasts released from the pump remained viable compared to control (n.s. by Student’s t-test). **(C)** When pumps were suspended in media adjacent to cells for 24 h, cell viability was not affected (n.s. by Student’s t-test).

Finally, we wanted to examine the device’s ability to deliver cells at defined time points, as well as the biocompatibility of the silicone device. We determined whether cells could be loaded into the device and released by the pumping mechanism by releasing 90 k fibroblasts equally into three well plates (30 k per well). Note that the residual volume in the pump resulted in an estimated ∼20 k cells per well with cells left in the device; for control wells the 90 k cells were distributed evenly across three wells (30 k per well). Visually, images demonstrated cell attachment following release and normal cell growth for 48 h post-release (Figure 7A). When the viability was measured at 48 h post-release, we found the control wells had an average of 46 k cells and the pump released wells had an average of 36 k cells. We attribute this to the lower number of initially seeded cells due to the dead volume (i.e., the volume that stays in the tubing/within the device after complete activation) in our device. Overall, the control wells showed 100% viability, whereas cells in the pump showed non-significant reduction in viability of 83.3% ± 14.4% (Figure 7B).

Next, to test the biocompatibility of the device, we placed sterilized pumps in the media of wells containing BJ fibroblast cells for 24 h and subsequently examined their viability. Groups had near identical–and non-significantly different–cell viabilities (control = 93 ± 2%; pump = 91% ± 9%) (Figure 7C). Collectively, these results show that the device is not cytotoxic in the conditions tested, and that the device can release viable cells.

## 4 Discussion

In this work we present a silicone soft robotic drug delivery device that has a reliable On-Off mechanism with user-defined triggering; controllable dosage release in terms of volume and timing; and the potential for longer-term treatments due to its ability to be refilled and biocompatibility. In addition, we demonstrated the capacity to deliver a range of therapeutics from the device, from small molecule drugs to viruses and cells. We also demonstrated its biocompatibility *in vitro*. While there are many systems developed for triggered drug delivery using a variety of stimuli, such as light, magnetic simulation, and ultrasound, the majority of these systems depend on affinity between the drug and carrier, and necessitate slow diffusion/degradation rates to limit release during the non-stimulated phase (Kearney and Mooney, 2013; Brudno and Mooney, 2015). As a result, most of these systems have a low, but non-zero, leakage or baseline release during the Off phase, and a less extreme difference between the On versus Off phase. Here, the mechanical control over release–whereby the hole in the silicone drug release chamber is sealed except in the presence of pressure exceeding its critical opening threshold–ensures there is no release until activation. We confirmed this in short term studies and over the course of 5 days when actuating to 80% of the opening pressure. Importantly, we demonstrate that this mechanism is independent of the therapeutic being delivered.

Our device separates the actuation reservoir from the therapy reservoir, which allows for refilling of the drug delivery chamber and separate pneumatic activation. There have been several other approaches and attempts for the use of soft robotics for drug delivery (e.g., Mendez KL. et al., 2024; Yang et al., 2024; Mair et al., 2021; Whyte et al., 2018). Other strategies for soft robotic delivery use non-pneumatic approaches by triggering release by magnetic stimulation or temperature changes. For example, a small microrobot with individual chambers used oscillating magnetic fields and orientation to pull on valves on the individual chambers to release drugs (Yang et al., 2024). Shape-memory polymers have also been used in soft robotic devices to entrap drug cargo and subsequently deform to expose the cargo in response to a temperature change (Heunis et al., 2023). In a previous iteration of the device in the present work, the ability to release drugs at specific timepoints to the surface of the heart, and in response to ECG signals was demonstrated (Mendez KL. et al., 2024; Mendez K. et al., 2024). Other examples of refillable soft-robotic systems use inlet and outlet drug ports in a rigid template with a magnetically-responsive deflective covering that pumps the drug out (So et al., 2014); a refilling port attached to a rate-controlling membrane system for sustained release (Williams et al., 2025); and an implanted programmable microinfusion system that can be refilled with ingested capsules that magnetically reload the robot (Iacovacci et al., 2021). While our system shares some functional similarities with these devices in terms of functionality, it offers a simple manufacturing approach combined with precise triggering. In the present version of the device, refilling ports were added to the device to allow for longer term delivery and/or loading and storage of labile drugs for shorter time periods prior to precise delivery. While the pneumatic approach requires tethering to the activation mechanism, here it allows for precise timing of delivery, and access to refilling.

Under the fabrication conditions used in this study, the device exhibits release in response to trigger pressures within the range of 28.5 mmHg–59.8 mmHg, which can be used in a single pulse full dose release (activating at 59.8 mmHg) or by doing step increases in that range (e.g., we demonstrated 28.5, 50, 59.8 mmHg steps here). Furthermore, the pump demonstrated no signs of leakage between testing intervals, or when it was activated to ∼80% opening pressure for 5 days, proving the effectiveness of its pressure-sensitive valve mechanism. Additionally, consistent volume release was observed over 12 consecutive repeats, demonstrating the device’s potential for temporally sensitive, or repeat-dosing applications. Based on our observations during experimentation, it was clear that an inconsistent residual volume is left in the device. Here, we used volume as the refill measure, since this relates to standard clinical practice (i.e., therapeutic delivery with syringe needles). However, this results in more variation among repeated release due to the fluctuating residual volume within the device over repeated cycles. In a second set of studies (Figure 2E) we implemented a strategy to equilibrate the pressure prior to actuation to account for fluctuations in residual volume. This reduced standard deviations to ∼6 µL suggesting that controlling pressure can increase dosage repeatability of the device. While there was variability in this repeated-triggering study, in future iterations of the device the loading pressure (as opposed to volume) can be controlled which should compensate for fluctuations in residual drug volume in the chamber after release. This ability to reload and trigger repeatedly from the device is a key new feature that is important for long term drug delivery needs.

In an effort to screen the device’s potential for broad applicability, we focused on testing a diverse range of therapeutics all of which have potential use in wound healing. The device successfully released: doxycycline (a small molecule antibiotic), AAV-eGFP (a virus capable of transducing skin cells), dexamethasone (a small molecule glucocorticoid capable of inducing circadian rhythms), and fibroblast cells. Importantly, in all the *in vitro* assays for these therapeutics, the dose released from the device performed equivalently to the manually delivered doses. Doxycycline is a known antibiotic that is used in wound treatment, and can even be suitable in combination treatments for some drug resistant *E. Coli* strains (Lai et al., 2016). It has previously been explored for wound healing in drug delivery strategies (Hu et al., 2023) and may additionally have direct (i.e., non-antibacterial) effects on wound healing, such as anti-oxidant, anti-inflammatory, and anti-scarring (Moore et al., 2020; Saliy et al., 2024). While we did not explore all these effects on wound healing here, we successfully demonstrated its antibacterial effects *in vitro* when released from our pump device.

From a direct signaling perspective, gene therapy is being increasingly explored as a treatment for challenging chronic wounds, and AAV has emerged as a preferred viral vector due its efficacy and non-integrating property (Shaabani et al., 2022; Karimzadeh et al., 2024; Agrawal et al., 2004; Ain et al., 2021). As a proof-of-concept, we delivered AAV encoding for green fluorescent protein, and demonstrated the ability of pump-released AAV to transduce cells equally effectively to manual delivery of the virus.

Next, we delivered dexamethasone successfully from the device, and showed the ability of fibroblast cells to close 2D scratch assays more effectively than non-treated groups. Our lab has become increasingly interested in circadian and biological rhythms, and their role in tissue regeneration (Srinivasan et al., 2022), and one of the key therapeutics used to synchronize circadian rhythms of cells in in vitro cultures is dexamethasone (Pagani et al., 2010; Izumo et al., 2006; Yang et al., 2022). For use in circadian medicine, precise timing of drug treatment is necessary, motivating the testing of the device in a potential circadian application. We note that dexamethasone is also known to have direct effects on fibroblasts, including enhanced proliferation (Li et al., 1998). While future work will fully delineate whether it is a direct effect of dexamethasone, or the induction of a circadian rhythm in these cells, addition of dexamethasone to cultures using the pump accelerated 2D wound closure. This was consistent with others that attributed enhanced wound closure following dexamethasone pulsing to circadian rhythm synchronization (Ngo et al., 2019; Beta et al., 2022; Tu et al., 2020; Nakazato et al., 2023).

Finally, we were able to successfully load fibroblasts into the pump and release them into culture. As fibroblasts are critical cells in wound healing, various modified fibroblasts have been tested in wound healing (e.g. Maione et al., 2015; Shams et al., 2022). The ability to release fibroblasts, and for fibroblast viability to be unaffected by the device’s preseroutnce points to the favorable biocompatibility properties of the device. Overall, these studies demonstrate the potential for this device to be used for precisely timed delivery of a wide range of therapeutics to skin wounds, and highlights its potential–with some modifications for implantation–for other therapeutic applications, such as cardiac, gastrointestinal, peritoneal, and subcutaneous delivery.

To fully realize the potential of this device, further studies and modifications are necessary. First, the long-term stability of various therapeutics, especially those with sensitive storage conditions or shorter shelf-lives (e.g., therapeutics that are normally stored at 4 °C). While this can partially be addressed by the reloading feature of this device, modifications to the therapeutics themselves can be considered, such as carrier materials or excipients. A portable pump system is also necessary for practical translation. As this iteration of the device is planned for use in skin wound healing, it will be superficially placed over the wound, with minimal contact with the wound environment. As there is some dynamic motion, the device would need a rigid housing or backing to protect it during its use. This would be designed to prevent pressure spikes that would cause accidental overdose or leakage of the device. The current actuation pressure of the device (28.5 mmHg) could be further tuned if needed to ensure a sufficient safety margin. Additionally, the refillable feature of this device means the device does not need to be loaded with large volumes all at once, helping to mitigate the concern of other non-refillable drug delivery devices. Notably, in a previous iteration of the device, we did implant a similar but non-refillable version in both rats and pigs, where it was used to read electrocardiograms and deliver an antiarrhythmic agent (amiodarone) in rats, and a chronotropic agent (epinephrine) in pigs (Mendez KL. et al., 2024). While these were relatively short-term studies, no immune response, device biofouling, or interference with cardiac activity due to implantation was observed. We have also implanted other silicon devices for longer periods (up to 14 days) and–while a standard foreign body response with a fibrous capsule was observed–there was no infection, tissue necrosis, or toxic leaching (Horvath et al., 2018). Finally, further detailed drug studies and *in vivo* testing are necessary. For example, an additional question that needs to be addressed is whether the loaded therapeutics can permeate the skin. In most wound scenarios the skin is significantly more permeable in the initial stages, however, select therapeutics may need co-delivery with permeability enhancers to achieve full efficacy (Junker et al., 2013; Hmingthansanga et al., 2022).

Here, we investigated the capabilities of a refillable silicone pump for precisely timed on/off drug delivery. We successfully validated the delivery mechanisms of this soft robotic device, demonstrating its versatility with various small molecules and biologics, as well as its biocompatibility for potential implantable applications. Overall, the pump facilitates repeatable therapeutic administration, while ensuring bioactivity, biocompatibility, and precise dosing across varying *in vitro* models.

## Data Availability

The raw data supporting the conclusions of this article will be made available by the authors, without undue reservation.
