# Supplementary material for: Refillable silicone pump with precise switching for timed therapeutic delivery
Source: Front Bioeng Biotechnol. 2025 Sep 11;13:1649771. doi: 10.3389/fbioe.2025.1649771 (PMC12460454; doi:10.3389/fbioe.2025.1649771)
Supplement: Supplementary file 1 [file DataSheet1.pdf]

Refillable Silicone Pump with Precise Switching for Timed Therapeutic Delivery

Naaz Thotathil[1 †], Dr. John J. Amante[1 †], Micah Wingell[1], Grace W. Hutter[2,3], Ultan Fallon[2,3], Yiling Fan[2,3], Dr. Keegan Mendez[2,3], Dr. Ellen Roche[2,3], Dr. Cathal J. Kearney*[1]

[1] Kearney Lab, Department of Biomedical Engineering, University of Massachusetts Amherst, Amherst, Massachusetts, USA

[2] Roche Lab, Department of Mechanical Engineering, Massachusetts Institute of Technology Cambridge, Massachusetts, USA

[3]Roche Lab, Institute for Medical Engineering and Science, Massachusetts Institute of Technology, Cambridge, Massachusetts, USA

*** Correspondence:**
Corresponding Author
ckearney@umass.edu

Supplemental Information

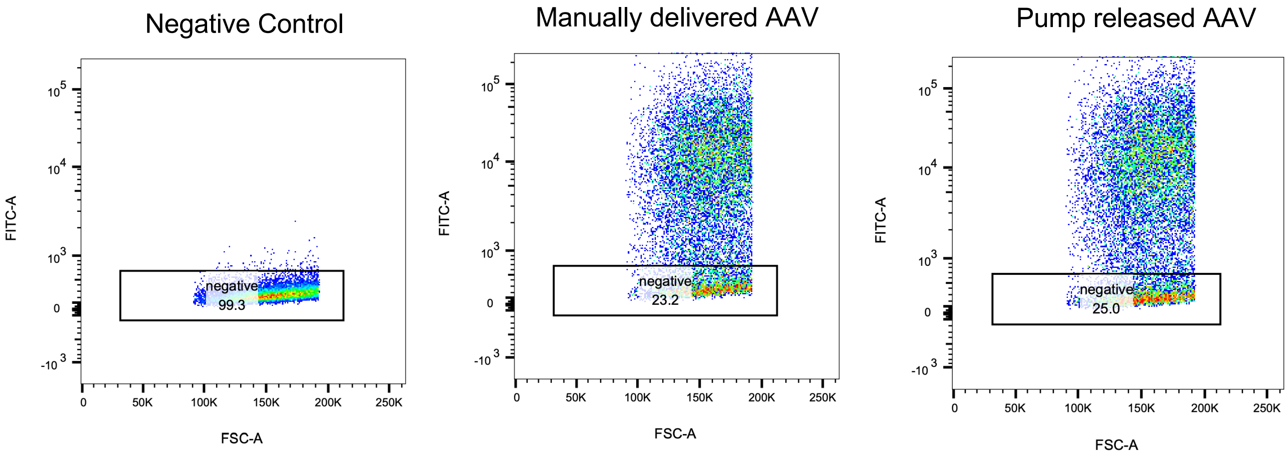

Figure S1. Sample data and gating strategy for flow cytometry data in Figure 4.

This is a provisional file, not the final typeset article
